# Metacognitive interventions in text production and working memory in students with ADHD

**DOI:** 10.1186/s41155-017-0081-9

**Published:** 2018-02-07

**Authors:** Nelba Maria Teixeira Pisacco, Yasmini Lais Spindler Sperafico, Jacqueline Raquel Bianchi Enricone, Luciano Santos Pinto Guimarães, Luis Augusto Rohde, Beatriz Vargas Dorneles

**Affiliations:** 1 Departamento de Educação, Universidade Estadual Ponta Grossa (UEPG), Praça Santos Andrade 01, Sala 113, CEP, Ponta Grossa, PR 84010-919 Brazil; 2 Universidade Federal Rio Grande do Sul (UFRGS), Porto Alegre, Brazil; 3 Universidade Regional Integrada do Alto Uruguai e das Missoes (URI), Erechim, Brazil; 4 Grupo de Pesquisa e Pós-Graduação at Hospital de Clinicas de Porto Alegre, Porto Alegre, Brazil

**Keywords:** Metacognition, Intervention, Attention-deficit hyperactivity disorder, Text production, Working memory

## Abstract

This study compared the effects of two metacognitive interventions on writing, working memory (WM), and behavioral symptoms of students with attention-deficit/hyperactivity disorder (ADHD). The disorder was clinically diagnosed by a multidisciplinary team according to DSM-IV criteria. The first approach consisted of a combined intervention in text production and WM while the second focused only on WM. Participants were 47 students from the fifth to ninth grades of two public elementary schools in Porto Alegre (Brazil), randomized to one of the two interventions groups. Writing and WM were assessed before, immediately after, and 3 months after the interventions. The results suggest that both interventions contributed to improving behavior and school performance, whereas only the combined intervention increased the overall quality of narrative text, organization of paragraphs, and denouement.

## Background

The high risk of low school performance associated with attention-deficit hyperactivity disorder (ADHD) is well established (American Psychiatric Association, 2014; DuPaul, Gormley, & Laracy, 2012; Sibley, Altszuler, Morrow, & Merrill et al., 2014). Writing seems to be the most impaired and the least studied skill in this group of children (Mayes & Calhoun, 2006; Dorneles et al., 2014). Moreover, a study showed that written expression skills affect academic performance in other areas much more than do the intensity of ADHD symptoms, comorbidity with oppositional defiant disorder, use of medication, reading ability, and intelligence (Molitor, Langberg, & Evans, 2016b).

The literature, albeit scarce, provides evidence of poor writing performance among ADHD students in childhood (Re, Pedron, & Cornoldi, 2007; Miranda, Soriano, & Baixauli, 2011), adolescence (Molitor et al., 2016a; DeBono et al., 2012), and young adults (Kim, Lee, & Lee, 2013; Miranda, Baixauli, & Colomer, 2013; Semrud-Clikeman, 2012). The writing of children and young people with ADHD is shorter, contains more errors, and is of poorer quality in terms of adequacy, structure, grammar, and lexicon, compared to that of their typically developing peers, and these difficulties affect different age groups (Re et al., 2007). The quality of the texts is worse even when they know the basic rules of writing, compared to peers without ADHD (Re & Cornoldi, 2010).

The behavioral symptoms of ADHD and/or neuropsychological and cognitive deficits, among which working memory (WM) is included (Sarver, Rapport, Kofler, & Friedman, 2015; Willcutt, Pennington, Olson, Chhabildas, & Hulslander, 2005), may contribute to poor performance in writing expression, as writing involves more than the simple transcription of thoughts and concepts and requires the involvement of a large number of highly complex cognitive processes such as essay planning, the production of ideas, their organization, the transcription, and the final revision, as highlighted by Re et al. (2007).

The cognitive approach to text composition began in the 1980s with Hayes and Flower, who proposed text production, involving long-term memory, the environment, and cognitive processes of planning, translation, and revision (Berninger, Whitaker, Feng, Swanson, & Abbott, 1996). Research on the role of WM in writing, conducted since the 1990s, has shown the processing capabilities of WM to be heavily involved in the coordination and alternation between writing processes (Olive, 2012). The WM is a temporary attention-regulated storage system that sustains our capacity for complex thought, such as language, planning, and problem-solving. It consists of four components: central executive, phonological loop, visuospatial sketchpad, and episodic buffer (Baddeley, 2012).

Considering the cognitive processes involved in composition, the study by Miranda et al., (2011) compared the performance of children with ADHD to those with typical development in written narrative texts. The results indicated that in the text of children with the disorder, the planning process was ineffective, compromising structure, cohesion, and coherence. Their narratives had fewer words and phrases, were less complex, and had a greater number of morphosyntactic, spelling, and presentation errors, indicating poorer performance in the translation process. Lack of revision or its ineffectiveness contributed to children’s failure to detect and correct formal errors and improve content. Difficulties in writing narratives may persist after elementary school, since the performance of young adults with ADHD in narrative texts is often poorer than that of their peers (Miranda et al., 2013).

The low quality of texts produced by ADHD students (Re et al., 2007; Re & Cornoldi, 2010; Kim et al., 2013; Miranda et al., 2011), the high prevalence of this disorder in childhood and adolescence (Polanczyk, Lima, Horta, Biederman, & Rohde, 2007), and the scarcity of publications on the topic underscore the clear need for further research on the development and implementation of interventions designed to facilitate the writing processes of students with this disorder (Miranda et al., 2013; Semrud-Clikeman, 2012; Molitor et al., 2016b). In this context, the question is: what interventions can contribute to improving the writing performance of students with ADHD?

Any intervention needs to consider how ADHD affects learning. Deficits in different WM components associated with ADHD (Martinussen, Hogg-Johnson, & Tannock, 2005) and poor performance in various writing skills (Graham, Fishman, Reid, & Hebert, 2016; Alloway, Gathercole, Kirkwood, & Elliott, 2009), as well as the relation between cognitive and metacognitive processes involved in text production and WM (Olive, 2012; Olive, Kellog, & Piolat, 2008; Berninger et al., 1996), should be considered when planning an intervention.

Notably, there are two foci of interventions that could contribute to this purpose. Considering the evidence of the relation between writing and WM and deficits in ADHD, interventions that aim to increase WM capacity could eventually improve performance in areas such as writing, which demand high WM levels. Yet, several WM intervention studies among children with ADHD have demonstrated improvement in WM. However, few have reported transfer effects on academic performance (Holmes et al., 2010; Klingberg, 2010; Gray et al., 2012; Gropper, Gotlieb, Kronitz, & Tannock, 2014). Moreover, the few studies that presented positive effects on WM capacity with transfer to school performance (Klingberg et al., 2005; Witt, 2011) have been criticized for focusing on WM training while neglecting central executive training (Morrison & Chein, 2011; Rapport, Orban, Kofler, & Friedman, 2013). Another criticism refers to the fact that the beneficial effects were specific and momentary, i.e., they were neither maintained in the long term nor generalized to other contexts, suggesting the need for broader programs or for the combination of general and specific interventions in the skills to be improved (Melby-Lervåg & Hulme, 2013; Rapport et al., 2013).

On the other hand, interventions focusing on writing processes that provide the writer with greater automaticity would relieve the overload on WM, because when a student automatizes the writing processes, less attention is required and the demand on working memory diminishes while writing performance improves. Among the interventions focusing on the cognitive processes of text production, the self-regulated strategy development (SRSD) instruction model developed by Harris and Graham in the 1990s (Graham, McKeown, Kiuhara, & Harris, 2012) is of particular note. The results and applications of their study have been revised several times (Harris, Graham, & Adkins, 2015; Harris, Graham, & Mason, 2008). The impact of SRSD on the writing quality produced by students with learning disabilities has been demonstrated (Graham, Harris, & McKeown, 2013).

SRSD combines the teaching of writing processes through explicit instruction in general writing strategies with specific strategies of the text genre and the development of self-regulation strategies, including the establishment of goals, self-assessment, self-instruction, and self-reinforcement (Harris, Friedlander, Saddler, Frizzelle, & Graham, 2005). The strategies are developed in an interactive and individualized manner, and the responsibility for writing is gradually delegated to the students (Harris et al., 2008).

The SRSD model addresses the WM deficits that are commonly found in students with ADHD (Jacobson & Reid, 2010; Molitor et al., 2016a; Miranda et al., 2013). In that model, the explicit teaching of strategies is designed to contribute to (a) reducing the demands on WM during the writing of a text, when used in an automated manner; ( b) developing a repertoire of flexible strategies to deal with academic tasks, which are scarce in children with ADHD; (c) improving focus and effort with the use of self-regulation strategies; and (d) setting goals, keeping goals in mind, and directing the behavior toward achieving them (Harris et al., 2015). This approach has been demonstrated to be effective in improving the quality of different text genres among students with ADHD (Reid & Lienemann, 2006; Jacobson & Reid, 2010; La Paz, 2001; Lienemann & Reid, 2008). However, a review article by Reid, Hagaman, and Graham (2014)found only 12 studies with ADHD students, totaling 27 participants. Although the interventions demonstrated significant effects, the limitations of the studies have to be taken into account: the small number of writing strategies tested, the small sample sizes, application solely in the context of special education and mostly to individuals, and the fact the results were measured immediately after the interventions, with no control of the long-term effect.

The methodological characteristics of SRSD, described above, demonstrate the metacognitive focus of this intervention model, in accordance with the four aspects of metacognition initially proposed by Flavell (1979): metacognitive knowledge (comprises of sensitivity to the self, to the task and to the strategy and the knowledge of those three elements), metacognitive experiences, objectives, and actions or strategies. Use of metacognition in WM interventions was encountered in the studies by Nunes (Nunes, Evans, Barros, & Burman, 2011 and Nunes, Barros, Evans, & Burman, 2014), who found metacognitive intervention to be effective in improving WM; however, the intervention was not investigated in specific groups of children with ADHD. This proposed intervention deserves to be further investigated as positive effects were found for metacognitive training with children with ADHD (Asli, Faramarzi, Arefi, Farhadi, & Fakkar, 2014). Thus, the central hypothesis of this study is that we will find greater improvement regarding specific text production skills and behavioral symptoms in students with ADHD when using a combined intervention rather than using a WM intervention alone.

No metacognitive interventions were found to have the dual focus, i.e., on WM skills and on specific text production skills. Therefore, this study proposes an innovative approach that considers the cognitive processes involved in metacognitive interventions in WM and in written expression. The main aim is to compare the effects of two metacognitive interventions on writing, school performance, WM, and behavioral symptoms of elementary school students with ADHD. One intervention combines WM training with specific text production skills, while the other focuses only on WM.

## Method

### Design

This experimental comparative intervention research analyzed measures obtained at three different time points: before, at the end, and 3 months after the intervention. The sample was randomized into two parallel groups: the combined intervention group (IG-Combined) and the WM intervention group (IG-WM).

### Ethical aspects

The research project was approved by the Research Ethics Committee of the Hospital de Clínicas de Porto Alegre. The study was conducted after obtaining authorization from the schools and adherence of teachers and after an informed consent form was signed by the students’ legal guardians and the students. Individual cases that were excluded from the sample but showed some ADHD signs or writing deficits were reported to the guardians. After the end of the study, the results were presented to the students, guardians, and schools and the Attention Deficit/Hyperactivity Program of the Hospital de Clínicas de Porto Alegre (ProDAH/HCPA) referred the participants for new clinical evaluations to determine the need for continued care.

### Participants and procedures

**Fig. 1:**
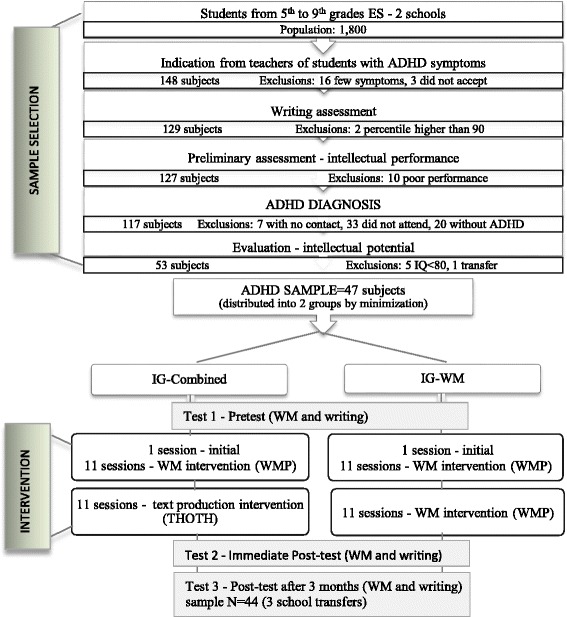
Flowchart of research stages. IG-Combined: combined intervention group, IG-WM: WM intervention group, WMP: Working Memory Program, WM: Working Memory, THOTH: Trabalhando com Habilidades de Organização de Textos Harmônicos [Working with Harmonic Text Organization Skills]

The sample was selected out of a population of approximately 1800 students from two public schools in Porto Alegre, capital city of Brazil’s southernmost state. Both schools, covered elementary education, were similar in curricular proposals, number of students, geographic location (downtown area), and socioeconomic characteristics of the population served (lower middle economic class). Sample selection, evaluation, and intervention were carried out at the participating schools (Fig. 1).

The sample consisted of 47 students from the fifth to ninth grades of elementary school, with mean age of 13.07 years (SD = 1.78). The inclusion criteria were Portuguese as main language; estimated IQ equal to or higher than 80, according to WISC-IV (Wechsler, 2013), and ADHD diagnosis, according to the DSM-IV criteria (American Psychiatric Association, 2002). The exclusion criteria were intellectual, sensory, or motor disabilities; autistic spectrum disorder, bipolar mood disorder, and depression in comorbidity with ADHD; and alphabetic or spelling phase of writing.

### Sample selection: instruments and procedures

The evaluation was carried out by external evaluators, consisting of educational psychologists, psychologists, and psychiatrists from the ProDAH/HCPA multidisciplinary team, researchers, and doctoral students with experience in the evaluation of children and adolescents with mental disorders.

The Portuguese version of the SNAP-IV (Mattos, Pinheiro, Rohde, & Pinto, 2006), validated for Brazilian samples and largerly used in the country (Caye, Machado, & Rohde, 2017), was applied to the teachers by education experts to identify probable cases of ADHD among students. It is a questionnaire formulated according to the DSM-IV criteria for ADHD diagnosis (American Psychiatric Association, 2002) that features 18 symptoms (nine related to inattentiveness and nine associated with hyperactivity/impulsivity) arranged on a Likert scale. Positive symptoms are those scored as quite a bit and very much. SNAP-IV was applied twice, before and soon after the intervention, in order to determine changes in the intensity of symptoms. On both occasions, the questionnaires were filled out by the same teacher.

The Balanced Dictation test (Moojen, 2011), composed of 50 words, was applied to determine the performance in alphabetic-spelling writing, since autonomy over writing of words is required for text production. The principal criterion used in selecting the words in the test was the frequency of the letters in the Portuguese language. So that, the most common letters and combinations of letters appeared more frequently (Moojen, 2011).

The selection of this test was based on the possibility of collective application; its scope to assess the fifth to ninth elementary school grades and the validation scores that were obtained from a population with socioeconomic characteristics similar to those of the sample, public school students from low middle class families. The test was used in other Brazilian studies: Franca, Wolff, Moojen, and Rotta (2004) used the test to highlight the acquisition of oral language as a predictive factor for the development of spelling; Moura, Cielo, and Mezzomo (2008)used the test to analyze mistakes in Portuguese writing of bilingual German-Portuguese children; Engelmann and Ferreira (2009) used the test to highlight the relationship between learning difficulties and auditory processing disorder in second grade students; Dorneles et al. (2014) used the test to evaluate the writing of ADHD students; and Basso et al. (2017) used it to evaluate the writing of adults with developmental dyslexia.

When applying the dictation, the evaluator described the activity, following the test instructions (Moojen, 2011, p. 82). In the correction, one point was assigned to every mistake made. Errors are classified into four types: phonological, simple grammatical spelling, complex grammatical spelling, and irregular phonological relation. The measures considered in the analysis were total number of mistakes in dictation and spelling accuracy obtained by the percentage of correct words. Students with a percentile higher than 90 were excluded because they did not present alphabetic writing required for text production.

A preliminary cognitive assessment was carried out to exclude cases of intellectual deficit by applying Raven’s colored progressive matrices—special scale—to subjects aged under 12 years (Angelini, Alves, Custódio, Duarte, & Duarte, 1999) and general scale (Raven, 2003) to students older than 12 years. In this study, results below the 50th percentile on the special scale and below the 10th percentile on the general scale were considered to have intelectual déficit and were excluded from the sample to keep the variable “intellectual level” as homogeneous as possible. All students with ADHD excluded from the sample were directed toward an appropriate special service.

In order to diagnose ADHD and comorbidities, the psychiatrists conducted the clinical evaluation in accordance with the DSM-IV criteria. The information was obtained from the children’s and adolescents’ parents through a semi-structured interview (Schedule for Affective Disorders and Schizophrenia for School-Aged Children—Epidemiologic Version [K-SADS-PL]) applied individually. According to the diagnostic criteria, ADHD was classified as combined, inattentive, or hyperactive/impulsive. The psychologists applied the vocabulary and cubes subtests of the Wechsler Intelligence Scale—WISC-IV (Wechsler, 2013) individually to evaluate IQ, and an IQ below 80 was an exclusion criterion.

The teachers indicated 148 students by means of the SNAP-IV, and after exclusions (Fig. 1), the sample was composed of 47 subjects, who were randomized into two groups (Table 1) by minimization, using the QMinim^1^ software. The following three factors were used: educational level (correlated with age *p* < 0.001), IQ, and school attended. In the stage of deferred post-tests, 3 months after the end of the interventions, the sample consisted of 44 participants, because two subjects from the IG-WM and one subject from the IG-Combined groups were transferred to different schools.

**Table 1:** Sample characteristics

		IG-Combined		IG-WM	
		Mean	SD	Mean	SD
Age		13.1	1.8	13.0	1.8
IQ		100	11.5	98.3	10.1
		N	(%)	*N*	(%)
Sex	Male	18	75	16	69.6
	Female	6	25	7	30.4
ADHD	ADHD-I	14	58.3	11	47.8
	ADHD-HI	2	8.3	1	4.3
	ADHD-C	8	33.3	11	47.8
Comorbidity	ODD	10	41.7	9	39.1
	CD	0	0.0	2	8.7
	SAD	1	4.2	1	4.3
	GAD	0	0.0	3	13.
	Phobias	2	8.4	4	17.3
	PTSD	1	4.2	0	0
	Epilepsy	1	4.2	0	0
	No comorbidity	11	45.8	9	39.1
Use of medication		4	16.7	4	17.4

*ADHD-I* predominantly of the inattentive type, *ADHD-HI* predominantly of the hyperactive/impulsive type, *ADHD-C* combined type, *ODD* opposition defiant disorder, *CD* conduct disorders, *GAD* generalized anxiety disorder, *SAD* separation anxiety disorder; *PTSD* post-traumatic stress disorder

### Evaluation of expressive writing and WM: procedures, instruments, and measures

The evaluation was performed three times: prior to the interventions (T1), immediately after the end of interventions (T2), and 3 months after the end of interventions (T3). The same instruments were used by external evaluators at T1, T2, and T3. At the time of application, the evaluators did not know the type of intervention in which subjects participated, and at the time of correction of the tests, they also did not know which stage of the evaluation the subjects were in (T1, T2, or T3).

The following instruments were used to assess WM:

- The backward digit span (Backward DS) subtest of the Wechsler scales (Wechsler, 2013) consists of seven lists of digits, spoken at an even pace by the evaluator that should be remembered in reverse order. This subtest evaluates executive control over immediate retention of verbal information.
- Backward spatial span (Backward SS): This test evaluates how recent visuospatial information is handled (Shiels Jr et al., 2008). It is a computer adaptation of the Corsi Block-Tapping Task and of the Spatial Span subtest integrated into WISC-IV (Wechsler, 2013), which incorporates characteristics of CANTAB’s Spatial Span Task, provided by Psychology Software Tools, Pittsburgh-PA (Shiels et al., 2008). It is similar to the Digits Span Subtest in the presentation of progressive sequences of stimuli and by requiring responses in reverse order, but it differs as to the stimuli, which are visuospatial.
- Rey Auditory Verbal Learning Test (RAVLT): It is a standardized test translated into Portuguese (Malloy-Diniz, Fuentes, Abrantes, Lasmar, & Salgado, 2010), used by Martins and Ortiz (2009) and Nobre et al. (2013) to evaluate phonological loop and episodic buffer (Martins & Ortiz, 2009; Nobre et al., 2013). It consists of a list of 15 words (list A) read by the examiner at a 1-s interval. This procedure is repeated five consecutive times and each presentation is followed by the request of oral enunciation of the words recalled by the participant (A1, A2, A3, A4, and A5). After completion of the five tests, a second list of interference (list B) is read; participants are asked to enunciate the words from list B. Immediately after this distracting task, individuals are asked to say the words they recall from list A (A6). After 20 to 30 min, they are asked again to enunciate the words from list A (A7). The total score is determined by the number of words correctly memorized at each attempt.

The following instruments were employed to evaluate written expression:

- Balanced Dictation test (Moojen, 2011), applied in the sample selection;
- Narrative Text Production Task (NTPT)—participants were asked to focus their attention on a short story that would be read. After reading the story, the evaluator read the header contained in the task: “Now it is your turn to tell a story, by writing a narrative. You may continue telling the story that has been read or create another story with the theme ‘on the way to school’.” It was made clear that the time limit to complete the task was 30 min. We provided no support material and no instruction on the use of any strategy to plan or revise the text.

The texts were coded with random numbers and were corrected by two external evaluators (an educational psychologist and a teacher of Portuguese, both with graduate degrees and extensive experience in elementary school education), who were unaware of the objective of this study, of the evaluation stage, and of the identity and education level of participants. The evaluators participated in a workshop on text correction for guidance on the criteria and registration of the evaluation.

The level of textual articulation was used as a measure of overall performance in text production, and the score was attributed according to hierarchical categories 1 to 4, as proposed by Costa and Boruchovitchb (2009, p. 175–176). The different types of text indicators were selected and grouped based on the processes of planning, translation, and revision, as described in the Appendix, according to the criteria employed by Miranda et al. (2011).

Discrepant results among evaluators were analyzed by the first author, who had the power to make the final decision. The text was used for detection of probable errors in the counting of quantitative variables, and the mean score attributed by the two evaluators was used for qualitative or counting variables with subjective interference (narrative elements and level of textual articulation). After some adjustments, we organized the variables that originated from calculations of the values of other variables.

- Classroom Student Performance Questionnaire (n.d): Small questionnaire developed for this research, applied to teachers soon after the end of the interventions, with the objective of obtaining information about participants’ classroom performance. It is composed of a scale of 10 items—four related to school performance and six related to behavior. The teacher should check one option for each item: little improvement, same as before, significant improvement, and highly significant improvement.

### Intervention programs and procedures

During the evaluation and intervention processes, the use of medication for ADHD was maintained, that is, the eight participants who used medication continued using it with no modification in its administration during the protocol under the supervision of their physicians.^2^ The remaining students were not medicated.

Two intervention programs were used, whose choice and organization took into account: (a) theoretical assumptions and previous evidence, (b) possible adaptations to the Brazilian population and to students with ADHD, (c) metacognitive approach, (d) use of a mixed method for the development of activities—partly developed individually and partly developed collectively (use of computer and other resources), and (e) application in the school environment.

The “Working Memory Program” (WMP), developed by the research group of Nunes (Nunes et al., 2014), at the University of Oxford, was used for improving WM. The WMP considers metacognitive skills as a means to develop self-control over automatic attention and over the use of information testing strategies in order to improve WM (Nunes et al., 2011). It involves the four WM components proposed by Baddeley (2012), consisting of three online games and five games with multimedia playback, each with seven difficulty levels, available at http://www.education.ox.ac.uk/ndcs. The games involve explicit teaching and training of metacognitive strategies to recall information, which increased progressively, and which, at times, needed to be remembered in the order they were presented (direct order), and, at other times, had to be recalled in the reverse order in which they appeared (indirect order). In this study, we used a version that was translated and adapted, upon permission from the authors, for use with Brazilian students.

The program for intervention in text production was developed with metacognitive strategies adapted from the SRSD model (Mason, Harris, & Graham, 2011; Harris et al., 2008), using the software *Trabalhando com Habilidades de Organização de Textos Harmônicos* [Working with Harmonic Text Organization Skills]—henceforth THOTH, and Thoth also refers to the Egyptian god who invented writing. The following features were included: THOTH software with set of strategies for individual work, a file for multimedia presentation with organization of guidelines for collective activities in each session, and a notebook with a set of guiding schemes and mnemonic codes. The intervention included the gradual transfer of responsibility from the mediator to the participants, both in each session and in the process as a whole.

THOTH is based on the teaching of three types of facilitators for the development of simple explicit strategies (memorization codes, guiding questions, and guiding schemes) involving the processes of planning, translation, and revision, which were translated into and adapted to Portuguese. Narrative text was the chosen genre as it is common to the different school grades and to the needs of students, as shown by the analysis of the elementary school curriculum and confirmed by the teachers of the participating schools.

#### IG-Combined and IG-WM interventions

The interventions occurred at the computer labs of the schools and were mediated by two educational psychologists, the researcher and a research assistant. Groups of four to eight students attended two to three weekly sessions that lasted 40 to 50 min, totaling 23 sessions over a period of approximately 3 months. In case of absence, the student participated in the activities on another occasion. Throughout the process, participants of both groups received collective guidelines, worked individually and in pairs, rehearsed and shared and discussed strategies.

The interventions were carried out in two stages (Fig. 1). The first stage was the same for both groups, beginning with the initial session and then 11 sessions on WM, developed with the WMP. In the second stage, the IG-WM still focused on WM while the IG-Combined focused on text production, with the use of THOTH.

#### Data analysis

Agreement between evaluators was determined by the *t* test for paired samples and by Pearson’s correlation coefficient for the variable “level of textual articulation” by the PABAK index.^3^ The evaluators obtained similar evaluations for most variables. The lowest agreements were for the categorical variable “level of textual articulation” (51.5% (*p* < 0.001) and for four quantitative variables: narrative elements (difference of − 0.67; *p* < 0.001), planning errors (difference of − 0.43; *p* < 0.05), presentation errors (difference of − 0.88; *p* < 0.05), and percentage of revision (difference of 3.73; *p* < 0.001). Before proceeding to the other analyses, the disagreements were adjusted by calculating the mean between the values given by evaluators to the categorical variable or by revising the count of quantitative variables.

The comparison between groups and time (group × time interaction) was made by generalized estimating equations (GEE). The normal distribution variables, tested by Shapiro-Wilk normality test, were estimated using an identity-link function. Variables with outcomes based on counting were analyzed using a Poisson distribution with log-link function. The proportion of the binary variable was estimated using a binary distribution with logit. For all models, we used an unstructured working correlation matrix and a robust estimator covariance matrix. The post hoc Bonferroni test was applied to significant factors. In an unstructured covariance matrix, there are no constraints. Each variance and each covariance is estimated uniquely from the data. This results in the best possible model fit, because each variance and covariance value is very close to what the data reflect.

Cohen’s effect size was computed for comparisons of the evaluation times of the groups, for the variables in which significant differences were found, and for the deltas of the evaluation times in the groups of the variable “level of textual articulation”. For the Classroom Student Performance Questionnaire, the groups were compared using the Mann-Whitney test as the questionnaire has only one measure and showed an assymetric distribution. We used the SPSS software, version 18, for the statistical analyses. The significance level was set at 0.05.

## Results

The results for written expression and WM are described considering the performance of the two intervention groups at the three time points for different measures, determining the group × time interactions and the effect size of interventions.

**Fig. 2:**
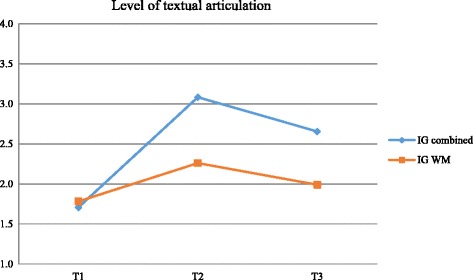
Level of textual articulation for IG-Combined and IG-WM

Table 2 also shows the significant results of writing processes. In the planning process, intervention group and time interaction was significant for narrative elements. The results with significant differences related to the translation process were as follows: group and time interaction was significant for words/clause (IG-WM was better at T1) and paragraph (IG-WM was better at T1 and IG-Combined improved at T2 and remained so at T3); significant differences in time indicated that both groups improved similarly after the intervention and that improvements were not maintained for text length, morphosyntactic errors, and presentation errors after 3 months. In the revision process, statistically significant differences were not found in the revision of formal aspects (IG-Combined rose from 11.6 to 20% and ended with 21.9, while IG-WM started with 9.7%, increased to 13.5%, and ended with 12.2%, with moderate effect size at T2 and T3) and time × group interaction was significant for content revision, and in total revision—improvement of content was more significant from T1 to T2 and it decreased between T2 and T3 but it was maintained compared to T1, whereas for total revision, both groups increased the percentage of revision, with the best performance obtained by IG-Combined, and improvement was maintained at T3, as shown in Fig. 2.

**Table 2:** Results of GEE and effect size for written expression measures of NTPT with significant differences

Variables	Time	Mean score								ES	*p* value		
		IG-Combined			IG-WM			General			Group	Time	Interaction
		Mean		SE	Mean		SE	Mean	SE				
Textual articulation^a^	1	*1.7*	*aA*	*0.1*	*1.8*	*aA*	0.2	1.8	0.11	0.09	0.009	< 0.001	*0.002*
	2	*3.1*	*bA*	*0.2*	*2.3*	*bB*	0.2	2.7	0.12	1.0			
	3	*2.7*	*cA*	*0.2*	*2.0*	*abB*	0.2	2.3	0.12	0.8			
	General	2.5		0.12	2.0		0.14						
Elements of the narrative^a^	1	4.6		0.3	4.3		0.2	*4.5a*	*0.2*	*0.22*	*0.018*	*< 0.001*	0.448
	2	6.0		0.2	5.2		0.3	*5.6b*	*0.2*	*0.83*			
	3	5.7		0.2	4.9		0.2	*5.3b*	*0.2*	*0.69*			
	General	*5.4*	*A*	*0.2*	*4.8*	*B*	*0.2*						
Denouement^b^	1	0.8	aA	0.09	0.61	aA	0.1	*0.68*	*0.07*	0.3	< 0.001	*< 0.001*	*< 0.001*
	2	1	bA	0	0.87	aA	0.07	*1*	*0*	0.5			
	3	1	bA	0	0.85	aB	0.08	0	0	3.4			
	Geral	0.9		0	0.8		0.04						
Planning errors^a^	1	3.0		0.7	2.7		0.6	*2.8*	*0.4*	0.1	0.646	*< 0.001*	0.112
	2	2.0		0.4	2.9		0.5	*2.4*	*0.3*	0.4			
	3	1.2		0.3	1.3		0.3	*1.2*	*0.2*	0.03			
	General	1.9		0.3	2.1		0.4						
Text extension^a^	1	63.1		12.5	62.4		9.7	*62.7*	*7.9*	0.01	0.685	*< 0.001*	0.822
	2	89.3		7.7	86.6		9.2	*87.9*	*6.0*	0.06			
	3	65.5		8.3	56.9		7.7	*61.1*	*5.7*	0.2			
	General	71.7		8.6	67.5		6.1						
Morphosyn-tactic errors^a^	1	3.5		0.5	4.7		0.6	*4.1*	*0.4*	0.4	0.243	*0.024*	0.699
	2	5.1		0.7	5.8		0.8	*5.4*	*0.5*	0.2			
	3	3.8		0.5	4.2		0.6	*4.0*	*0.4*	0.2			
	General	4.1		0.4	4.8		0.5						
Presentation errors^a^	1	7.7		1.0	9.4		1.2	*8.5*	*0.8*	0.3	0.137	*0.001*	0.317
	2	11.2		1.5	18.0		4.9	*14.2*	*2.1*	0.4			
	3	8.0		1.1	7.9		1.3	*7.9*	*0.9*	0.01			
	General	8.8		0.8	11.0		1.3						
Words/Clauses^c^	1	5.5	aA	0.2	6.0	aB	0.2	*5.7*	*0.2*	0.6	0.580	*0.265*	0.030
	2	5.6	aA	0.2	5.3	bA	0.1	*5.5*	*0.1*	0.5			
	3	5.6	aA	0.3	5.7	abA	0.3	*5.6*	*0.2*	0.09			
	General	5.6		0.1	5.7		0.2						
Paragraph^c^	1	1.8	aA	0.1	1.4	aB	0.1	*1.6*	*0.1*	0.6	< 0.001	0.002	*0.031*
	2	2.5	bA	0.2	1.5	aB	0.1	*2.0*	*0.1*	1.3			
	3	2.1	abA	0.2	1.5	aB	0.1	*1.8*	*0.1*	0.8			
	General	2.1		0.1	1.5		0.1						
Punctuation^c^	1	1.8		0.1	1.6		0.2	*1.7*	*0.1*	0.3	0.002	*0.681*	0.401
	2	2.0		0.1	1.5		0.1	*1.8*	*0.1*	0.8			
	3	2.0		0.1	1.5		0.1	*1.7*	*0.1*	0.9			
	General	1.9	A	0.1	1.5	B	0.1						
Revision^c^	1	11.8		2.6	8.37		1.3	*10.1*	*1.5*	0.3	0.004	*< 0.001*	0.198
	2	23.8		3.6	13.1		2.4	*18.5*	*2.1*	0.7			
	3	23.2		4.2	11.9		2.2	*17.6*	*2.3*	0.7			
	General	19.6	A	2.5	11.1	B	1.5						

Time: 1: pretest; 2: immediate posttest; 3: deferred post-test; general: mean between evaluation times. Different lowercase letters represent statistically different mean values, by comparing time and setting group. Different uppercase letters represent statistically different mean values, by comparing groups and setting times

*IG-Combined* combined intervention group, *IG-WM* Working Memory Intervention Group, *SE* standard error, *ES* effect size

^a^Poisson distribution

^b^Binary logit distribution

^c^Normal distribution

**Fig. 3:**
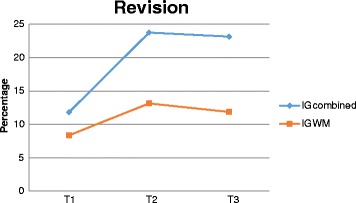
Percentage of revision for IG-Combined and IG-WM

The group that received the combined intervention performed better in textual articulation, measure used to assess the overall quality of text production, with highly significant group × time interaction and large effect size between groups at T2 and T3. Both groups improved, but the results show significant differences in favor of IG-Combined, as illustrated by Fig. 3

The mean level of textual articulation for IG-Combined and IG-WM did not differ in the pretest and the greatest difference occurred in the immediate post-test. The computation of Cohen’s effect size, with relation to time deltas, resulted in a large effect size between the groups (ΔT1-T2 (ES = 0.99) and of ΔT1-T3 (ES = 0.85)) and within the groups (IG-Combined ΔT1-T3 = 1.00 and IG-WM ΔT1-T3 = 0.19), indicating greater effect and greater maintenance of quality for IG-Combined.

Other measures concerning writing were obtained by employing the Balanced Dictation test. Significant differences in time indicated progressive reduction of errors (T1 = 21.4, T2 = 17.8, and T3 = 16.0 for IG-Combined and T1 = 22.3, T2 = 18.3, and T3 = 17.0 for IG-WM) and an increase in spelling accuracy (64.75 to 69.08 and 73.22 for IG-Combined and 64.87 to 67.91 and 71.29% for IG-WM) immediately after the interventions and 3 months thereafter.

Table 3 shows GEE results for WM and intensity of symptoms with significant differences. Group × time interaction in Backward SS showed better IG-WM performance in the pretest. ADHD symptoms were measured by SNAP-IV at T1 and T2, and group × time interaction was significant for hyperactivity/impulsivity severity, with reduction in IG-Combined.

**Table 3:** Results of GEE and effect size for measures of Working Memory and ADHD symptoms

Variables	Time	Mean score										*p* value		
		Combined			WM			General			ES	Group	Time	Interaction
		Mean		SE	Mean		SE	Mean		SE				
RAVLT A7^a^	1	9.3		0.7	8.7		0.6	*9.0*	*A*	*0.5*	0.19	0.552	*< 0.001*	0.327
	2	10.7		0.7	10.7		0.5	*10.7*	*B*	*0.4*	0.01			
	3	11.0		0.5	10.3		0.7	*10.6*	*B*	*0.4*	0.27			
	General	10.3		0.5	9.9		0.5							
DI-backward^a^	1	8.8		0.5	9.3		0.4	*9.0*	*A*	*0.3*	0.27	0.209	*0.049*	0.753
	2	8.2		0.4	8.9		0.5	*8.6*	*B*	*0.3*	0.34			
	3	8.5		0.6	9.5		0.5	*9.0*	*ab*	*0.4*	0.38			
	General	8.5		0.4	9.3		0.4							
SS-backward^c^	1	*4.7*	*aA*	*0.2*	*5.3*	*aB*	*0.2*	5.0		0.1	0.59	0.794	0.759	*0.014*
	2	*5.2*	*aA*	*0.2*	*5.0*	*aA*	*0.2*	5.1		0.1	0.23			
	3	*5.0*	*aA*	*0.2*	*4.9*	*aA*	*0.2*	4.9		0.2	0.20			
	General	5.0		0.2	5.0		0.2							
Inattention^c^	1	1.9		0.11	1.9		0.1	*1.9*	*A*	*0.09*	0.00	0.928	*< 0.001*	0.896
	2	1.4		0.12	1.4		0.2	*1.4*	*B*	*0.1*	0.04			
	General	1.7		0.10	1.7		0.1							
Hyperactivity/impulsivity^c^	1	*1.2*	*aA*	*0.2*	*1.0*	*Ba*	*0.2*	1.1		0.1	0.33	0.692	0.003	*0.035*
	2	*0.8*	*bA*	*0.2*	*0.9*	*Aa*	*0.2*	0.8		0.1	0.14			
	General	1.0		0.2	0.9		0.2							
Total symptoms^c^	1	1.6		0.1	1.4		0.1	*1.5*	*A*	*0.08*	0.24	0.816	**<** *0.001*	0.194
	2	1.1		0.1	1.2		0.2	*1.1*	*B*	*0.09*	0.11			
	General	1.3		0.1	1.3		0.1							

Time: 1: pretest; 2: immediate posttest; 3: deferred post-test; general: mean between evaluation times. Different lowercase letters represent statistically different mean values, by comparing time and setting group. Different uppercase letters represent statistically different mean values, by comparing groups and setting times

*IG-Combined* combined intervention group, *IG-WM* Working Memory Intervention Group, *SE* standard error, *ES* effect size

^a^Poisson distribution

^b^Binary logit distribution

^c^Normal distribution

There were no significant differences between the groups (Mann-Whitney *p* > 0.05) for any of the items of school performance and behavioral aspects reported by teachers in the Classroom Student Performance Questionnaire. Considering the total sample, the percentage of students who showed significant or very significant improvement in performance was 55.3% in mathematics, 63.8% in writing, 47.8% in other subjects, and 61.7% in reduction of errors in assessments and activities. As for behavior in the classroom, considering the total number of students in both groups, there was significant or very significant improvement: 53.2% in organization, 51.1% in meeting deadlines, 59.6% in persistence in performing activities that require more cognitive effort, 54.3% in attention to explanations and classroom activities, 42.6% in agitated behavior, and 51.1% in compliance with rules.

## Discussion and conclusions

An innovative intervention process was carried out among students with ADHD with the aim of comparing two interventions using a metacognitive approach. While both programs used self-regulation strategies, such as self-control of attention and self-monitoring of behavior, their focus differed—one intervention focused on working memory and writing and the other only on working memory. Our central aim was to compare the effects of the two interventions on specific text production skills and different aspects of elementary school performance of students with ADHD.

The results show group × time interaction at the level of articulation and measures of writing processes, suggesting better performance in the group receiving the combined intervention. The planning process had significant differences for denouement, a narrative element, and the translation process was significant for words/clause (IG-WM was better at T1) and paragraph (IG-WM was better at T1 and IG-Combined improved at T2 and remained so at T3). Statistically significant differences were not found in the revision of formal aspects (IG-Combined went from 11.6 to 20% and ended with 21.9, while IG-WM started with 9.7%, rose to 13.5%, and ended with 12.2%), and time and group interaction was significant for content revision and in total revision. Content improvement was more significant from T1 to T2; there was reduction from T2 to T3, but improvement was maintained compared to T1 whereas the percentage of revision increased in both groups, with the best performance observed in IG-Combined and improvement maintained at T3, as shown in Fig. 2.

Improvement in written textual articulation after intervention with metacognitive strategies had been previously observed by Costa and Boruchovitch (2009) in a study with Brazilian children with typical development. Although no previous study has been conducted with students with ADHD in Brazil, evidence of improved text quality among students with the disorder after intervention in the SRSD model was found in a study from the USA (Reid et al., 2014). Similar to the results of the present research, an increase in structural elements in writing and in text length after interventions based on the same model among students with ADHD was also observed in the review by Reid et al. (2014). However, in the aforementioned review of studies, no evaluation was conducted on the maintenance of the effects for longer periods, and the effects were not compared between different interventions. Great difficulty in the revision process among students with ADHD was reported by Rodriguez et al. (2009) and Miranda et al. (2011). While those studies did not include an intervention, the difficulties described by them may help us to explain why in the present study, although combined intervention contributed to increasing revision, correction attempts continued to be insufficient in relation to the total text errors after the intervention.

WM measurements did not show significant differences between the two groups: WM and combined intervention. The study by Nunes et al., 2014 found favorable results in these WM measures in deaf children after the intervention, with the same program used for both groups in the first stage of intervention and in the second stage for IG-WM. Improvement in WM measures in students with ADHD after the intervention was found in studies that employed other programs (Klingberg et al., 2005; Gray et al., 2012; Gropper et al., 2014). However, when comparing the results of this study with those cited above, the following must be taken into consideration: unlike this study, the other studies did not compare two groups that received WM interventions and the evaluation was conducted with tasks similar to those which were trained. Thus, the differences between the results may be explained, in part, by methodological differences; therefore, the difficulty of comparing the studies should be emphasized.

The severity of ADHD symptoms was investigated by means of SNAP-IV before and immediately after the interventions, suggesting a significant reduction in the intensity of hyperactivity/impulsivity symptoms for IG-Combined. We can suppose that the reduction could be linked to the improved inhibitory control developed as a consequence of the writing training with metacognitive focus. School performance and behavioral aspects reported by the Classroom Student Performance Questionnaire showed improvement in behavioral symptoms (organization, meeting deadlines, persistence in activities that require more cognitive effort, attention in the classroom, agitated behavior, and compliance with rules) and school performance (mathematics, writing and other subjects, reduction of errors in assessments and activities) in about 50% (*N* = from 11 to 17 depending on the category) of the participants of both groups. Beneficial effects of computer programs for WM training, such as reduction of symptoms caused by the disorder, were also found in other studies (Gropper et al., 2014; Gray et al., 2012). Metacognitive interventions in WM strategies and writing process strategies can improve the writing of students with ADHD, which can be effective in the major ADHD symptoms, as shown in the study by Asli et al. (2014). In summary, the results suggest the combined intervention improved some aspects of writing, without compromising the improvement of WM, despite the lesser intensity of specific training compared with the intervention only in WM (12 vs. 23 sessions), and reduced the severity of hyperactivity symptoms. The greatest improvements were observed: (a) in the writer’s skill of integrating the basic elements of a narrative with the ideas intended to be expressed in the text, as measured by the level of articulation; (b) in the structure of the narrative text, described by narrative elements; and (c) in improved paragraph structure and use of punctuation.

The results of this study must be considered within the context of its limitations: the sample was selected from only two public schools that may not be representative of the Brazilian population of students with ADHD; no formal evaluation of comorbidity with learning disorders was made; use of handwriting in the evaluation of texts, differently from the training conducted in the intervention, which employed computer in the writing; collective evaluation of writing may have interfered with text production because of ADHD symptoms; no comparison with either a non-intervention control group or an intervention focused only on text production strategies. School grade and the time the school teachers spent on writing narratives in daily activities were not controlled, variables that must be taken into account in future studies.

Despite the limitations, the contributions and implications of the study are noteworthy. The results show that psychoeducation interventions carried out within the school context may contribute to improving the writing performance of students with ADHD. The evidence obtained from teachers’ reports indicates transfer effects from interventions in reducing the intensity of ADHD symptoms, in improving school performance, and in improving behavior in the classroom. Improvement in writing and WM performance can also be considered a transfer effect, since it results from distinct activities in relation to those trained in the interventions. Finally, our findings suggest that the combined intervention in text production and WM strategies was more effective in improving the production of narrative texts by students with ADHD than an intervention focusing only on WM strategies. Given that access to special services is limited for many children with ADHD, the most important pratical educational implication of this research is that it demonstrates the effectiveness of conducting interventions that improve writing and diminish the symptoms of ADHD in schools.

## Appendix

**Table 4:** Description of NTPT correction criteria according to writing process

NTPT correction criteria		
Indicators by process		Score
Planning	Narrative elements**	1 point per element present in the text: introduction of subject matter, characters, ambiance, problem or purpose, action, denouement and emotions; (range=0–7).
	Temporal sequence error*	1 point per each alteration in the chronological order of events.
	Content errors/digressions*	1 point per one of off-topic statements.
	Cohesion errors*	1 point per each incomplete and/or ambiguous reference by inadequate use or absence of connectives.
	Planning errors	Sum of sequence, content, and cohesion errors.
Translation	Number of words*	1 point for words of the text.
	Number of clauses*	1 point per clauses (1 point per unit composed by subject and predicate that included a personal form of a verb).
	Words per clause*	Division of number of words by number of clauses.
	Number of sentences	1 point per sentence.
	Number of compound sentences	1 point per compound sentence (more than one clause).
	Syntactic complexity*	Division between compound sentences and number of sentences.
	Morphosyntactic errors*	1 point per error (agreement of gender, number, or between subject and verb; in the order of words in sentences; omissions, substitutions or additions of functional words [such as articles, prepositions, adverbs]).
	Spelling errors*	1 point per spelling error (there can be more than one error in a word).
	Correct words	1 point per word with no spelling errors.
	Absolute spelling accuracy	Percentage of number of correct words in relation to number of words.
	Relative spelling accuracy	Percentage of number of words spelled correctly and number of words that are not monosyllables.
	Presentation errors*	1 point per error (erasures, illegible words, inadequacy as to capitalization, spacing and separation of words, etc.).
	Paragraphing (organization of text content into paragraphs)	Considering the text in its entirety, assign: 1 for no division of paragraphs; 2 for inadequate paragraphing; 3, partially adequate paragraphing; and 4 for adequate punctuation (range=1–4).
	Score	Considering the text in its entirety, assign: 1 for no punctuation; 2 for inadequate punctuation; 3 for partially adequate punctuation; and 4 for adequate punctuation (range=1–4).
Revision	Formal revisions*	1 point per each rewriting of illegible words, correction of punctuation, and correction of spelling.
	Content revisions*	1 point per each addition, exclusion, shift, and transformation of word, sentence, or clause in attempting to improve the content.
	Formal revision	Percentage of formal revisions in relation to total formal errors
	Content revision	Percentage of content revisions in relation to total content errors
	Revision*	Percentage of formal revisions and content revisions in relation to total revisions.

Chart 1: Description of NTPT correction criteria according to writing process

Source: Adaptation of the criteria proposed by Miranda, Soriano and Baixauli (2011)* and Costa and Boruchovitchb (2009)**

## Footnotes

1 Online version of MinimPy (Saghaei & Saghaei, 2011) available at http://qminim.sourceforge.net/demo/.

2 Information on medication use during the evaluations and interventions was obtained from parents by written communication and included data on the physicians who prescribed the medication. 6 students used methylphenidate and 2 used risperidone. Statistically significant differences were not found in the results of medicated and unmedicated participants,

3 Resource available at http://www.singlecaseresearch.org/calculators/pabak-os .

## Authors’ information

Nelba M. T. Pisacco is a PhD in Education at the School of Education at Universidade Federal Rio Grande do Sul (UFRGS), graduated in Pedagogy, and is a professor at Universidade Estadual de Ponta Grossa (UEPG), Ponta Grossa, Brazil, and a researcher of the ADHD Outpatient Program at Hospital de Clínicas de Porto Alegre, Brazil.

Yasmini L. S. Sperafico is a PhD in Education at the School of Education at Universidade Federal Rio Grande do Sul (UFRGS), graduated in Mathematics, and is a member of the ADHD Outpatient Program at Hospital de Clínicas de Porto Alegre, Brazil.

Jacqueline Raquel Bianchi Enricone is a PhD in Education at the School of Education at Universidade Federal Rio Grande do Sul (UFRGS), graduated in Psychology, and is a professor at Universidade Integrada do Alto Uruguai e das Missões (URI), Erechim, Brazil, and a researcher of the ADHD Outpatient Program at Hospital de Clínicas de Porto Alegre, Brazil.

Luciano Santos Pinto Guimarães is a Master in Epidemiology at Universidade Federal do Rio Grande do Sul (UFRGS), graduated in Mathematics and in Statistics, and is a statistical analyst in the Group of Research and PostGraduation (GPPG) and a member of the Research Ethics Committee of the Hospital de Clínicas de Porto Alegre, Brazil.

Luís A. Rohde is a PhD in Psychiatry at Universidade Federal Rio Grande do Sul (UFRGS),graduated in Medicine, is a full professor of Psychiatry at Universidade Federal Rio Grande do Sul (UFRGS) and a director of the ADHD Outpatient Program at Hospital de Clínicas de Porto Alegre, Brazil.

Beatriz V. Dorneles has a pós-doctoral studies at Oxford University, is a full professor of Education in the Post-Graduate Programme of the School of Education, is a PhD in Education at Universidade Federal Rio Grande do Sul (UFRGS) and a member of the ADHD Outpatient Program at Hospital de Clínicas de Porto Alegre, Brazil.
